# Crystal structure of poly[bis­(μ-2-amino-4,5-di­cyano­imidazolato-κ^2^ *N* ^1^:*N* ^3^)-*trans*-bis­(*N*,*N*′-di­methyl­formamide-κ*O*)cadmium]

**DOI:** 10.1107/S2056989015014516

**Published:** 2015-08-12

**Authors:** Jin-Li Zhu, Guo -Qing Jiang, Xiao-Qing Guo, Yan-Feng Tang, Miao Wang

**Affiliations:** a College of Chemistry and Chemical Engineering, Nantong University, Nantong 226019, People’s Republic of China; b School of Textile and Clothing, Nantong University, Nantong 226019, People’s Republic of China

**Keywords:** crystal structure, 2-amino-4,5-di­cyano­imidazole, metal–organic framework, cadmium coordination polymer, hydrogen bonding

## Abstract

In the title structure, [Cd(C_5_H_2_N_5_)_2_(C_3_H_7_NO)_2_]_*n*_ or [Cd(adci)_2_(DMF)_2_]_*n*_, the Cd^2+^ ion is located on a twofold rotation axis and is six-coordinated in a CdN_4_O_2_ manner by four imidazole N atoms of four symmetry-related 2-amino-4,5-di­cyano­imidazolate (adci) anions in the equatorial plane and by two O atoms of symmetry-related *N*,*N*-di­methyl­formamide (DMF) ligands in axial positions. The adci^−^ anions bridge adjacent Cd^2+^ ions [shortest Cd⋯Cd separation = 6.733 (3) Å] into a layered coordination polymer extending parallel to (001). The primary amino group and the non-coordinating cyano groups of adci^−^ anions are involved in hydrogen-bonding inter­actions with DMF ligands to stabilize the crystal structure.

## Abstract 2

The title compound, [Cd(C_5_H_2_N_5_)_2_(C_3_H_7_NO)_2_]_*n*_, is a two-dimensional coordination polymer extending parallel to (100). Notably, both the primary amino group and the cyano groups are involved in hydrogen-bonding inter­actions with DMF ligands to direct the assembly and stabilize the crystal packing.

## Chemical context   

Porous materials such as metal-organic frameworks (MOFs) combining advantages of both organic and inorganic components have emerged as a unique class of crystalline solid-state materials today due to their potential applications in gas adsorption and separation (Collins & Zhou, 2007 ▸), catalysis (Gu *et al.*, 2012 ▸) and analytical chemistry (Mondal *et al.*, 2013 ▸). As a branch of MOFs, zeolitic imidazolate frameworks (ZIFs), which are topologically related to inorganic zeolites, commonly reveal high thermal and chemical stability (Eddaoudi *et al.*, 2015 ▸). Bridging N-donor ligands such as 2-substituted 4,5-di­cyano­imidazole (dci) mol­ecules are often used to synthesize ZIFs (Sava *et al.*, 2009 ▸; Mondal *et al.*, 2014 ▸). In addition, the cyano group of dci can generate carboxyl­ate- (Orcajo *et al.*, 2014 ▸) or tetra­zole-based (Xiong *et al.*, 2002 ▸) ligands by *in-situ* ligand reactions.
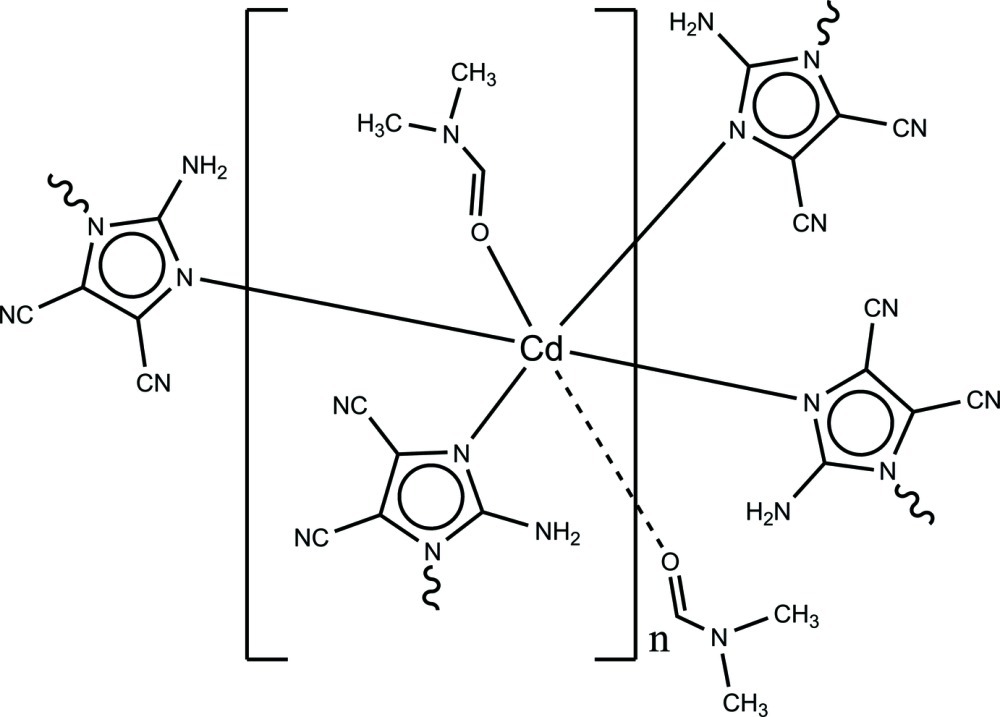


We chose a rigid planar ligand, *viz.* 2-amino-4,5-di­cyano­imidazole (adci), and Cd^2+^ that exhibits strong coordination capabilities for imidazolates, to prepare new metal-organic polymers and report here the structure of the title compound, [Cd(C_5_H_2_N_5_)_2_(C_3_H_7_NO)_2_]_*n*_, or [Cd(adci)_2_(DMF)_2_]_*n*_ (DMF is di­methyl­formamide), (I).

## Structural commentary   

**Figure 1:**
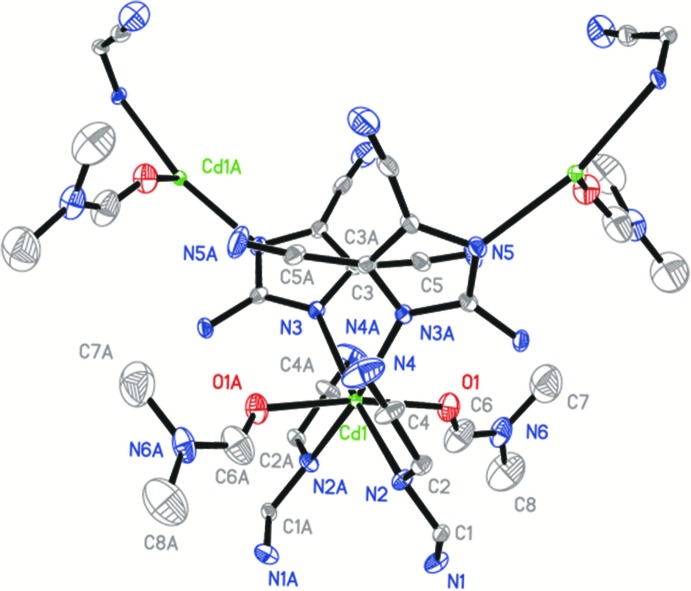
The coordination sphere around Cd^2+^ in the structure of (I), with displacement ellipsoids drawn at the 30% probability level. H atoms bonded to C and N atoms have been omitted for clarity. [Symmetry code: (A) 2 − *x*, *y*, 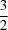 − *z*.].

**Figure 2:**
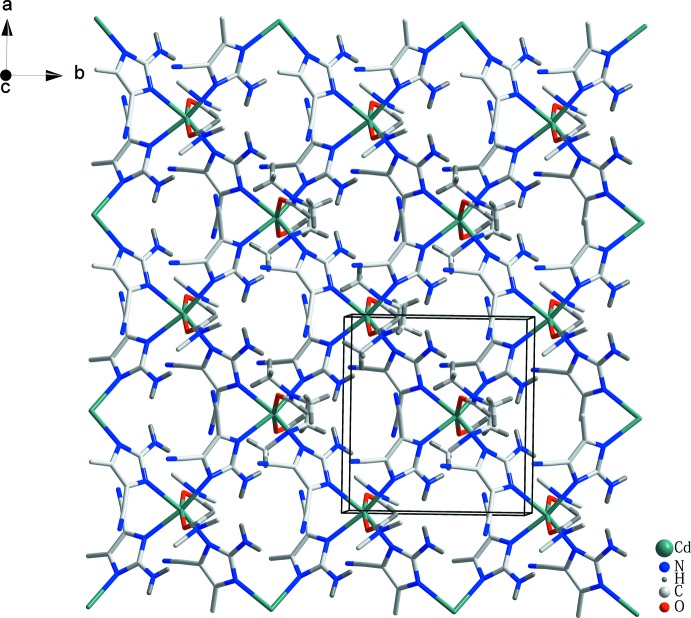
The two-dimensional network in the structure of (I), viewed perpendicular to the *ab* plane. Colour key: Cd steel, N blue, H grey, C light grey ande O red.

Complex (I) is a mononuclear cadmium coordination polymer, in which the central Cd^2+^ ion exhibits a tetra­gonally distorted octa­hedral coordination environment (Fig. 1 ▸). The asymmetric unit of (I) comprises one Cd^2+^ ion located on a twofold rotation axis, one 2-amino-4,5-di­cyano­imidazolate ion and one DMF ligand, both in general positions. The Cd^2+^ ion has an N_4_O_2_ coordination set defined by four N atoms of four symmetry-related adci^−^ anions in the equatorial plane and by two oxygen atoms of two symmetry-related DMF ligands in axial positions. The Cd—N bond lengths [2.339 (4) and 2.353 (4) Å] and Cd—O bond length [2.322 (4) Å] fall in normal ranges (Groom & Allen, 2014 ▸). Each adci^−^ anion bridges two adjacent Cd^2+^ ions in a bis-monodentate mode through two imidazole N atoms whereas the DMF mol­ecules serve as terminal ligands. Thus, four Cd^2+^ ions and four bridging adci^−^ ligands generate a square motif aligned parallel to (001), as shown in Fig. 2 ▸. The Cd⋯Cd distance along the edge of the square is 6.733 (3) Å, which is similar to previously reported structures (Li *et al.*, 2010 ▸; Wang *et al.*, 2010 ▸).

## Supra­molecular features   

**Figure 3:**
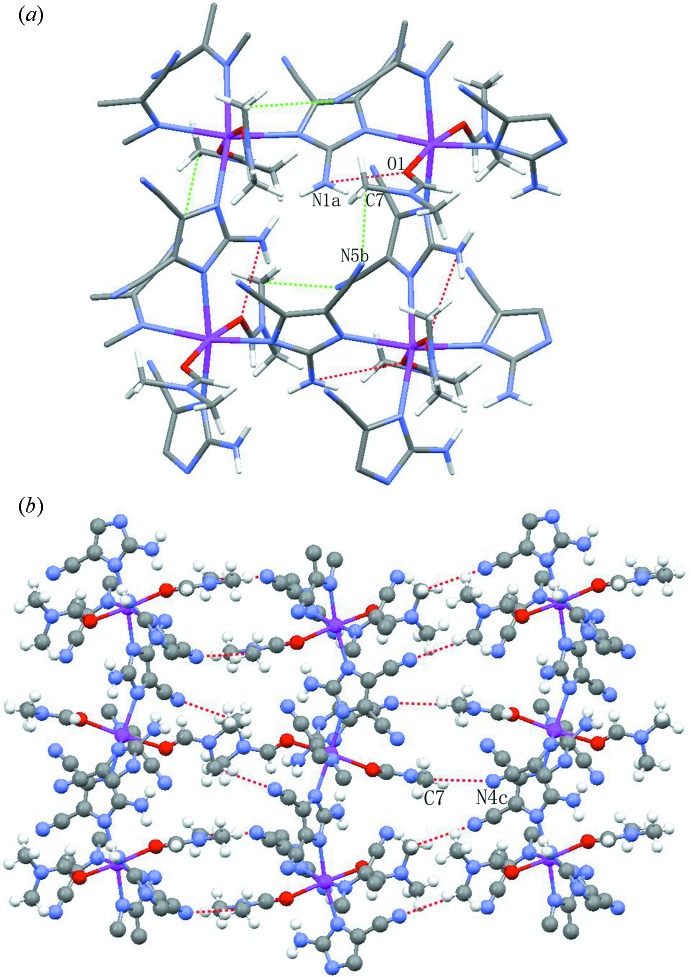
(*a*) View of two kinds of hydrogen bonds in the layers. Dashed lines represent C—H⋯N (green) and N—H⋯O (red) hydrogen bonds, respectively. (*b*) The crystal packing between the layers in the title structure. C—H⋯N hydrogen-bonding inter­actions are drawn as red dashed lines. [Symmetry codes: (*a*) 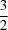 − *x*, −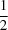 + *y*, *z*; (*b*) 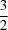 − *x*, 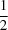 + *y*, *z*; (*c*) *x*, −*y*, −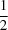 + *z*].

Complex (I) possesses various hydrogen-bonding inter­actions (Table 1 ▸). The amino group and the non-coordinating cyano N atoms are involved in hydrogen-bonding inter­actions with DMF ligands to stabilize the crystal structure. In the 2D metal-organic network, inter­molecular N1—H1*A*⋯O1 hydrogen bonds between the primary amine group of adci^−^ and the O atoms of an DMF ligand as well as C7—H7*C*⋯N5 inter­actions between the methyl C atoms of DMF and the non-coordinating N atoms of the cyano group of an adci^−^ anion play a crucial role in directing and stabilizing the assembly of the supra­molecular structure (Kim *et al.*, 2015 ▸; Sava *et al.*, 2009 ▸), as shown in Fig. 3 ▸ *a*. The layers are packed together by weak C7—H7*B*⋯N4 inter­actions, involving the methyl C atom of DMF and another N atom of a cyano group (Fig. 3 ▸ *b*). The lengths of these three hydrogen bonds fall in or approach the range (3.2–4.0 Å) of weak hydrogen-bonding inter­actions (Desiraju, 1996 ▸; Steed & Atwood, 2000 ▸).

## Database survey   

The cyano groups of the dci ligands exhibit a strong electron-withdrawing effect. Consequently, the formation of anionic species is relatively straightforward (Prasad *et al.*, 1999 ▸) and 4,5-di­cyano­imidazoles can be used in the preparation of coordination frameworks with different metal ions. However, reports on systems with 2-amino-4,5-di­cyano­imidazole, a novel rigid planar ligand with five potential coordination sites, are rather scarce. A search in the Cambridge Structural Database (Version 5.27, May 2014; Groom & Allen, 2014 ▸) for 4,5-di­cyano­imidazole revealed eleven complexes with 2-substituted 4,5-imidazole­dicarbo­nitrile ligands. An unprecedented SHG-active silver-containing MOF with a rare 10^3^ topology has been reported (Yang *et al.*, 2013 ▸), as well as the synthesis and fluorescent properties of a 3D heterometallic polymeric complex {[K[Cd(dci)_2_(H_2_O)_6_]Cl]}_*n*_ (Li *et al.*, 2010 ▸), and of {[Zn_2_(IMDN)_4_(H_2_O)_3_]·3H_2_O_3_}_*n*_ and [Co(IMDN)_2_(H_2_O)_2_]_*n*_ (Hu *et al.*, 2013 ▸) (IMDN is 2*H*-imidazole-4,5-dicarbo­nitrile) with chain structures. However, the coordination modes of the imidazoles in these complexes are different.

## Synthesis and crystallization   

Compound (I) was synthesized as follows: adci (0.0266 g, 0.2 mmol) and HNO_3_ (0.2 ml, 3.5 *M* in DMF) were mixed in 2 ml DMF. After stirring for 0.5 h, Cd(NO_3_)_2_·4H_2_O (0.0308 g, 0.1 mmol) in 6 ml methanol was added dropwise. The mixture was further stirred for another hour and then filtrated. The filtrate was kept at ambient temperature. After about three weeks, yellow block-shaped crystals of (I) suitable for single X-ray diffraction were obtained. Yield: 0.0224 g (43% based on Cd). FT–IR (KBr, cm^−1^): 3436, 3346, 2930, 2217, 1658, 1525, 1486, 1444, 1385, 1328, 1305, 1115, 675.

## Refinement   

Crystal data, data collection and refinement details are summarized in Table 2 ▸. Hydrogen atoms of the organic ligands were placed in idealized positions, with *d*(C—H) = 0.93 Å for *sp* ^2^-bound H atoms and *U*_iso_(H) = 1.2*U*_eq_(C), and *d*(C—H) = 0.96 Å for methyl H atoms and *U*_iso_(H) = 1.5*U*_eq_(C). H atoms of the amino group were located from a difference map and were refined with *d*(N—H) = 0.86 Å and *U*_iso_(H) = 1.2*U*_eq_(N).

## Supplementary Material

Crystal structure: contains datablock(s) global, I. DOI: 10.1107/S2056989015014516/wm5187sup1.cif

Structure factors: contains datablock(s) I. DOI: 10.1107/S2056989015014516/wm5187Isup2.hkl

CCDC reference: 1416545

Additional supporting information:  crystallographic information; 3D view; checkCIF report

## Figures and Tables

**Table 1:** Hydrogen-bond geometry (, )

*D*H*A*	*D*H	H*A*	*D* *A*	*D*H*A*
N1H1*A*O1^i^	0.86	2.45	3.187(6)	144
C7H7*C*N5^i^	0.96	2.68	3.429(12)	135
C7H7*B*N4^ii^	0.96	2.65	3.496(11)	148

Symmetry codes: (i) 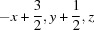; (ii) 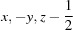.

**Table 2:** Experimental details

Crystal data	
Chemical formula	[Cd(C_5_H_2_N_5_)_2_(C_3_H_7_NO)_2_]
*M*_r_	522.83
Crystal system, space group	Orthorhombic, *P* *b* *c* *n*
Temperature (K)	296
*a*, *b*, *c* ()	9.8438(2), 9.1897(2), 22.8948(4)
*V* (^3^)	2071.10(7)
*Z*	4
Radiation type	Mo *K*
(mm^1^)	1.10
Crystal size (mm)	0.18 0.12 0.10

Data collection	
Diffractometer	Bruker SMART APEX CCD area detector
Absorption correction	Multi-scan (*SADABS*; Bruker, 2008 ▸)
*T*_min_, *T*_max_	0.854, 0.896
No. of measured, independent and observed [*I* > 2(*I*)] reflections	9497, 2386, 1741
*R*_int_	0.022
(sin /)_max_ (^1^)	0.650

Refinement	
*R*[*F* ^2^ > 2(*F* ^2^)], *wR*(*F* ^2^), *S*	0.042, 0.159, 1.07
No. of reflections	2353
No. of parameters	143
No. of restraints	96
H-atom treatment	H-atom parameters constrained
_max_, _min_ (e ^3^)	1.71, 0.66

Computer programs: *SMART* and *SAINT* (Bruker, 2008 ▸), *SHELXTL* (Sheldrick, 2008 ▸), *Mercury* (Macrae *et al.* (2006 ▸) and *DIAMOND* (Brandenburg, 2006 ▸).

## supplementary crystallographic information

### Crystal data

**Table:**

[Cd(C_5_H_2_N_5_)_2_(C_3_H_7_NO)_2_]	*F*(000) = 1048
*M**_r_* = 522.83	*D*_x_ = 1.677 Mg m^−^^3^
Orthorhombic, *P**b**c**n*	Mo *K*α radiation, λ = 0.71073 Å
Hall symbol: -P 2n 2ab	θ = 3.5–27.5°
*a* = 9.8438 (2) Å	µ = 1.10 mm^−^^1^
*b* = 9.1897 (2) Å	*T* = 296 K
*c* = 22.8948 (4) Å	Block, yellow
*V* = 2071.10 (7) Å^3^	0.18 × 0.12 × 0.10 mm
*Z* = 4	

### Data collection

**Table:**

Bruker SMART APEX CCD area-detector diffractometer	2386 independent reflections
Radiation source: fine-focus sealed tube	1741 reflections with *I* > 2σ(*I*)
Graphite monochromator	*R*_int_ = 0.022
phi and ω scans	θ_max_ = 27.5°, θ_min_ = 4.1°
Absorption correction: multi-scan (*SADABS*; Bruker, 2008)	*h* = −12→11
*T*_min_ = 0.854, *T*_max_ = 0.896	*k* = −11→11
9497 measured reflections	*l* = −29→26

### Refinement

**Table:**

Refinement on *F*^2^	Primary atom site location: structure-invariant direct methods
Least-squares matrix: full	Secondary atom site location: difference Fourier map
*R*[*F*^2^ > 2σ(*F*^2^)] = 0.042	Hydrogen site location: inferred from neighbouring sites
*wR*(*F*^2^) = 0.159	H-atom parameters constrained
*S* = 1.07	*w* = 1/[σ^2^(*F*_o_^2^) + (0.0999*P*)^2^ + 4.109*P*] where *P* = (*F*_o_^2^ + 2*F*_c_^2^)/3
2353 reflections	(Δ/σ)_max_ < 0.001
143 parameters	Δρ_max_ = 1.71 e Å^−^^3^
96 restraints	Δρ_min_ = −0.65 e Å^−^^3^

### Special details

**Table:**

Geometry. All e.s.d.'s (except the e.s.d. in the dihedral angle between two l.s. planes) are estimated using the full covariance matrix. The cell e.s.d.'s are taken into account individually in the estimation of e.s.d.'s in distances, angles and torsion angles; correlations between e.s.d.'s in cell parameters are only used when they are defined by crystal symmetry. An approximate (isotropic) treatment of cell e.s.d.'s is used for estimating e.s.d.'s involving l.s. planes.
Refinement. Refinement of *F*^2^ against ALL reflections. The weighted *R*-factor *wR* and goodness of fit *S* are based on *F*^2^, conventional *R*-factors *R* are based on *F*, with *F* set to zero for negative *F*^2^. The threshold expression of *F*^2^ > σ(*F*^2^) is used only for calculating *R*-factors(gt) *etc*. and is not relevant to the choice of reflections for refinement. *R*-factors based on *F*^2^ are statistically about twice as large as those based on *F*, and *R*- factors based on ALL data will be even larger.

### Fractional atomic coordinates and isotropic or equivalent isotropic displacement parameters (Å^2^)

**Table:**

	*x*	*y*	*z*	*U*_iso_*/*U*_eq_	
Cd1	1.0000	0.11224 (5)	0.7500	0.0180 (2)	
N1	0.8425 (4)	0.4650 (5)	0.71416 (18)	0.0327 (10)	
H1A	0.8043	0.5387	0.6978	0.039*	
H1B	0.9243	0.4416	0.7050	0.039*	
N2	0.8289 (4)	0.2699 (4)	0.78205 (17)	0.0269 (9)	
C4	0.7391 (6)	0.1135 (5)	0.8626 (2)	0.0360 (12)	
O1	0.9090 (4)	0.1297 (4)	0.65667 (17)	0.0449 (10)	
N3	1.1454 (4)	−0.0840 (4)	0.72898 (18)	0.0224 (8)	
C1	0.7732 (6)	0.3852 (5)	0.75474 (18)	0.0235 (10)	
C2	0.7281 (5)	0.2249 (5)	0.81951 (18)	0.0258 (9)	
C3	1.1166 (4)	−0.1874 (5)	0.6876 (2)	0.0234 (9)	
N6	0.9069 (5)	0.1975 (7)	0.5616 (2)	0.0516 (13)	
N5	0.8982 (5)	−0.2271 (6)	0.6291 (3)	0.0571 (15)	
N4	0.7418 (7)	0.0315 (6)	0.9000 (2)	0.0651 (17)	
C5	0.9921 (5)	−0.2038 (6)	0.6565 (2)	0.0308 (11)	
C6	0.9373 (9)	0.2022 (10)	0.6174 (3)	0.0700 (18)	
H6	0.9931	0.2801	0.6272	0.084*	
C8	0.9492 (13)	0.3011 (17)	0.5189 (5)	0.122 (3)	
H8A	0.9115	0.3947	0.5282	0.183*	
H8B	0.9178	0.2711	0.4811	0.183*	
H8C	1.0465	0.3073	0.5187	0.183*	
C7	0.8193 (12)	0.0771 (13)	0.5425 (5)	0.113 (3)	
H7A	0.8668	−0.0134	0.5472	0.169*	
H7B	0.7959	0.0902	0.5022	0.169*	
H7C	0.7381	0.0758	0.5657	0.169*	

### Atomic displacement parameters (Å^2^)

**Table:**

	*U*^11^	*U*^22^	*U*^33^	*U*^12^	*U*^13^	*U*^23^
Cd1	0.0143 (3)	0.0181 (3)	0.0217 (3)	0.000	0.00059 (15)	0.000
N1	0.023 (2)	0.032 (2)	0.043 (2)	0.0050 (18)	0.0117 (18)	0.0122 (19)
N2	0.0247 (19)	0.029 (2)	0.027 (2)	0.0072 (17)	0.0029 (15)	0.0036 (16)
C4	0.043 (3)	0.033 (3)	0.032 (2)	0.017 (2)	0.010 (2)	0.0043 (19)
O1	0.048 (2)	0.060 (2)	0.0270 (18)	0.0003 (19)	−0.0062 (17)	0.0052 (16)
N3	0.0155 (18)	0.024 (2)	0.0272 (18)	0.0032 (16)	0.0015 (16)	−0.0037 (17)
C1	0.022 (2)	0.024 (2)	0.024 (2)	0.0054 (17)	−0.0030 (16)	−0.0004 (16)
C2	0.029 (2)	0.026 (2)	0.023 (2)	0.0059 (19)	0.0019 (18)	0.0001 (17)
C3	0.021 (2)	0.021 (2)	0.028 (2)	−0.0009 (17)	0.0007 (18)	−0.0001 (17)
N6	0.052 (3)	0.076 (3)	0.027 (2)	0.014 (3)	−0.006 (2)	0.005 (2)
N5	0.036 (3)	0.069 (4)	0.067 (4)	−0.004 (3)	−0.019 (3)	−0.021 (3)
N4	0.100 (5)	0.054 (3)	0.041 (3)	0.030 (3)	0.022 (3)	0.018 (2)
C5	0.029 (3)	0.028 (3)	0.035 (3)	−0.0021 (19)	0.001 (2)	−0.008 (2)
C6	0.072 (4)	0.084 (4)	0.054 (3)	0.025 (4)	0.004 (3)	0.007 (3)
C8	0.143 (7)	0.138 (8)	0.085 (6)	0.023 (7)	0.016 (6)	0.036 (6)
C7	0.116 (7)	0.130 (7)	0.092 (6)	0.023 (6)	−0.024 (5)	−0.032 (6)

### Geometric parameters (Å, º)

**Table:**

Cd1—O1^i^	2.322 (4)	C1—N3^iii^	1.342 (7)
Cd1—O1	2.322 (4)	C2—C3^iii^	1.371 (6)
Cd1—N2^i^	2.339 (4)	C3—C2^ii^	1.371 (6)
Cd1—N2	2.339 (4)	C3—C5	1.426 (6)
Cd1—N3^i^	2.353 (4)	N6—C6	1.311 (9)
Cd1—N3	2.353 (4)	N6—C8	1.427 (13)
N1—C1	1.366 (6)	N6—C7	1.469 (12)
N1—H1A	0.8600	N5—C5	1.136 (7)
N1—H1B	0.8600	C6—H6	0.9300
N2—C1	1.347 (6)	C8—H8A	0.9600
N2—C2	1.375 (6)	C8—H8B	0.9600
C4—N4	1.141 (6)	C8—H8C	0.9600
C4—C2	1.426 (6)	C7—H7A	0.9600
O1—C6	1.154 (8)	C7—H7B	0.9600
N3—C1^ii^	1.342 (7)	C7—H7C	0.9600
N3—C3	1.371 (6)		
			
O1^i^—Cd1—O1	172.1 (2)	N3^iii^—C1—N1	123.0 (4)
O1^i^—Cd1—N2^i^	88.18 (14)	N2—C1—N1	122.3 (5)
O1—Cd1—N2^i^	86.91 (14)	C3^iii^—C2—N2	109.1 (4)
O1^i^—Cd1—N2	86.91 (14)	C3^iii^—C2—C4	124.4 (4)
O1—Cd1—N2	88.18 (14)	N2—C2—C4	126.3 (4)
N2^i^—Cd1—N2	103.5 (2)	C2^ii^—C3—N3	108.9 (4)
O1^i^—Cd1—N3^i^	95.71 (15)	C2^ii^—C3—C5	124.5 (4)
O1—Cd1—N3^i^	90.38 (15)	N3—C3—C5	126.6 (4)
N2^i^—Cd1—N3^i^	167.68 (14)	C6—N6—C8	125.3 (9)
N2—Cd1—N3^i^	88.42 (14)	C6—N6—C7	116.6 (8)
O1^i^—Cd1—N3	90.38 (15)	C8—N6—C7	118.0 (8)
O1—Cd1—N3	95.71 (15)	N5—C5—C3	173.8 (6)
N2^i^—Cd1—N3	88.42 (13)	O1—C6—N6	133.3 (9)
N2—Cd1—N3	167.68 (15)	O1—C6—H6	113.4
N3^i^—Cd1—N3	79.89 (19)	N6—C6—H6	113.4
C1—N1—H1A	120.0	N6—C8—H8A	109.5
C1—N1—H1B	120.0	N6—C8—H8B	109.5
H1A—N1—H1B	120.0	H8A—C8—H8B	109.5
C1—N2—C2	103.4 (4)	N6—C8—H8C	109.5
C1—N2—Cd1	129.4 (3)	H8A—C8—H8C	109.5
C2—N2—Cd1	122.0 (3)	H8B—C8—H8C	109.5
N4—C4—C2	174.4 (6)	N6—C7—H7A	109.5
C6—O1—Cd1	131.7 (6)	N6—C7—H7B	109.5
C1^ii^—N3—C3	103.9 (4)	H7A—C7—H7B	109.5
C1^ii^—N3—Cd1	132.5 (3)	N6—C7—H7C	109.5
C3—N3—Cd1	123.1 (3)	H7A—C7—H7C	109.5
N3^iii^—C1—N2	114.7 (4)	H7B—C7—H7C	109.5
			
O1^i^—Cd1—N2—C1	136.9 (5)	N2^i^—Cd1—N3—C3	−116.7 (4)
O1—Cd1—N2—C1	−36.8 (5)	N2—Cd1—N3—C3	78.0 (8)
N2^i^—Cd1—N2—C1	49.6 (4)	N3^i^—Cd1—N3—C3	59.4 (3)
N3^i^—Cd1—N2—C1	−127.3 (5)	C2—N2—C1—N3^iii^	−0.9 (5)
N3—Cd1—N2—C1	−145.5 (6)	Cd1—N2—C1—N3^iii^	153.2 (3)
O1^i^—Cd1—N2—C2	−73.1 (4)	C2—N2—C1—N1	178.5 (4)
O1—Cd1—N2—C2	113.1 (4)	Cd1—N2—C1—N1	−27.4 (7)
N2^i^—Cd1—N2—C2	−160.5 (4)	C1—N2—C2—C3^iii^	1.1 (5)
N3^i^—Cd1—N2—C2	22.7 (3)	Cd1—N2—C2—C3^iii^	−155.5 (3)
N3—Cd1—N2—C2	4.4 (9)	C1—N2—C2—C4	−174.0 (5)
O1^i^—Cd1—O1—C6	44.5 (6)	Cd1—N2—C2—C4	29.4 (6)
N2^i^—Cd1—O1—C6	−7.3 (6)	N4—C4—C2—C3^iii^	−35 (8)
N2—Cd1—O1—C6	96.3 (6)	N4—C4—C2—N2	140 (7)
N3^i^—Cd1—O1—C6	−175.3 (6)	C1^ii^—N3—C3—C2^ii^	−0.4 (5)
N3—Cd1—O1—C6	−95.4 (6)	Cd1—N3—C3—C2^ii^	172.2 (3)
O1^i^—Cd1—N3—C1^ii^	−34.6 (4)	C1^ii^—N3—C3—C5	179.5 (5)
O1—Cd1—N3—C1^ii^	140.3 (4)	Cd1—N3—C3—C5	−7.9 (7)
N2^i^—Cd1—N3—C1^ii^	53.5 (4)	C2^ii^—C3—C5—N5	−1 (6)
N2—Cd1—N3—C1^ii^	−111.8 (7)	N3—C3—C5—N5	179 (100)
N3^i^—Cd1—N3—C1^ii^	−130.4 (5)	Cd1—O1—C6—N6	164.9 (6)
O1^i^—Cd1—N3—C3	155.1 (4)	C8—N6—C6—O1	178.2 (9)
O1—Cd1—N3—C3	−30.0 (4)	C7—N6—C6—O1	−0.2 (13)

Symmetry codes: (i) −*x*+2, *y*, −*z*+3/2; (ii) *x*+1/2, *y*−1/2, −*z*+3/2; (iii) *x*−1/2, *y*+1/2, −*z*+3/2.

### Hydrogen-bond geometry (Å, º)

**Table:**

*D*—H···*A*	*D*—H	H···*A*	*D*···*A*	*D*—H···*A*
N1—H1*A*···O1^iv^	0.86	2.45	3.187 (6)	144
C7—H7*C*···N5^iv^	0.96	2.68	3.429 (12)	135
C7—H7*B*···N4^v^	0.96	2.65	3.496 (11)	148

Symmetry codes: (iv) −*x*+3/2, *y*+1/2, *z*; (v) *x*, −*y*, *z*−1/2.
